# Specific Aspects of SELEX Protocol: Different Approaches for ssDNA Generation

**DOI:** 10.3390/mps8020036

**Published:** 2025-04-06

**Authors:** Alexandr Garanin, Andrey Shalaev, Lidia Zabegina, Ekaterina Kadantseva, Tatiana Sharonova, Anastasia Malek

**Affiliations:** Subcellular Technology Lab, N.N. Petrov National Medical Research Center of Oncology, St. Petersburg 197758, Russia; garanin@niioncologii.ru (A.G.); shalaevav@niioncologii.ru (A.S.); zabeginalm@niioncologii.ru (L.Z.); kadanceva@niioncologii.ru (E.K.); sharonovatv@niioncolgii.ru (T.S.)

**Keywords:** aptamer, SELEX, PCR, single-stranded DNA, asymmetric PCR

## Abstract

Background: Synthetic DNA aptamers are a class of molecules with potential applications in medicine, serving as molecular sensors or ligands for targeted drug delivery. Systematic evolution of ligands by exponential enrichment (SELEX) is a technology for selecting functional aptamers that was first reported three decades ago and has been actively developed since. SELEX involves multiple iterations of two fundamental steps: (i) target affinity-based partitioning of aptamers from a random library and (ii) amplification of selected aptamers by PCR, followed by isolation of single-stranded DNA (ssDNA). SELEX protocols have diversified considerably, with numerous variations possible for each step. This heterogeneity makes it challenging to identify optimal methods. Comparative analysis of different approaches for the major stages of SELEX is therefore of considerable practical importance. Methods: Four widely used methods for ssDNA generation were performed in parallel: (a) PCR followed by digestion of the antisense strand with exonuclease lambda, (b) PCR with an extended primer followed by size-dependent strand separation using denaturing PAGE, (c) asymmetric PCR, and (d) asymmetric PCR with a primer-blocker. Results: The specificity, efficiency, reproducibility, and duration of each method were compared. Conclusions: Asymmetric PCR with a primer-blocker yielded the most favorable results.

## 1. Introduction

Aptamers are single-stranded oligonucleotides that adopt a defined three-dimensional structure, conferring upon them an affinity for a specific ligand [1]. They show great potential for use in diagnostics, drug delivery, and the treatment of certain diseases, especially cancer [2]. The process of functional aptamers development is known as SELEX (Systematic Evolution of Ligands by EXponential Enrichment). Since the SELEX principle was first proposed more than three decades ago [3], the procedure has been subject to numerous modifications and optimizations [4]. In general, SELEX involves incubating an excess of a randomized library of single-stranded DNA (ssDNA) with the target molecules, followed by purification of target-bound aptamers and their amplification. A single “incubation–separation–amplification” round is typically insufficient to select aptamers with high target affinity. The iterative SELEX process continues for several rounds until the library is sufficiently enriched for the desired aptamers. To proceed with each subsequent round, the double-stranded PCR products (dsDNA) must be converted to ssDNA. The efficiency of this conversion is crucial for the overall effectiveness of the SELEX procedure. Numerous approaches to generate ssDNA libraries have been proposed and successfully implemented; however, the optimal method remains undetermined.

Methods for generating ssDNA libraries can be broadly divided into two categories: (1) methods that separate ssDNA from the dsDNA product of conventional PCR and (2) ssDNA production via asymmetric PCR. The first category of methods is more extensive and widely used, employing various principles for denaturing dsDNA amplicons (e.g., high temperature or chemical denaturation) and a range of approaches for single-strand purification. For instance, a method involving PCR with an extended reverse primer (RV), which incorporated a spacer as a terminator (hexaethylene glycol (HEGL) and a 20-adenine nucleotide extension, i.e., poly(dA)_20_, followed by size-dependent chain separation using denaturing polyacrylamide gel electrophoresis (dPAGE), was proposed in 1995 [5]. This method is robust but labor-intensive. An alternative approach involves PCR with a biotin-labeled reverse primer (RV), followed by attachment of the dsDNA amplicon to streptavidin-coated beads and release of the ssDNA via alkaline or heat denaturation. Although technologically elegant, this method is limited by the inability to distinguish between desired products and byproducts, and by the potential for contamination of subsequent selection rounds with streptavidin [6,7,8]. Another popular approach for ssDNA generation is enzymatic cleavage of the complementary chain by lambda phage exonuclease [9,10]. In this approach, PCR is performed with a phosphate-labeled reverse primer (RV), and the phosphate-labeled strand subsequently undergoes enzymatic digestion. However, nucleases are not completely specific, and some of the non-phosphorylated strand may also be digested. Furthermore, any PCR byproducts lacking a phosphate group will be carried through to the selection stage and post-reaction purification. Moreover, the necessity of electrophoresis followed by gel purification of the ssDNA makes the procedure labor-intensive. In addition to the methods mentioned above, new approaches are continually being reported. One example is PCR with an acrydite-modified RV followed by polyacrylamide immobilization [11]. Another example is PCR with Texas Red-modified RV followed purification of the ssDNA from its complementary strand via ion pair reversed phase HPLC [12]. Thus, the first category of methods continues to be actively developed.

The second category of methods utilizes asymmetric PCR (A-PCR), a technique first described in 1988 [13]. It utilizes unequal primer concentrations for amplification, typically in a ratio ranging from 1:5 to 1:100. During the initial PCR cycles, while both primers remain available, amplification proceeds exponentially, generating dsDNA amplicons. Once the limiting reverse primer (RV) is depleted in the reaction mixture, only the remaining forward primer (FW) continues amplification, producing ssDNA amplicons. In the context of SELEX, asymmetric PCR was first proposed as a method for amplifying pre-selected individual clones [14]; subsequently, its application was extended to generating ssDNA directly during the amplification of enriched libraries [15,16]. However, the efficient application of asymmetric PCR requires careful optimization of numerous parameters, including primer ratios, PCR cycle number, annealing temperature, template concentration, Mg^2+^/dNTP concentrations, and Taq polymerase amount [17]. While optimized, this method is excellent for generating ssDNA from small amounts of template DNA; however, due to self-annealing of the single-stranded PCR products, it is prone to nonspecific amplification. Consequently, the number of PCR cycles must be adjusted iteratively throughout the SELEX procedure. Recently, a modification of the A-PCR method has been proposed to reduce nonspecific amplification: primer-blocked asymmetric PCR (PBA-PCR) [18]. Further improvement of A-PCR may involve modifying the length or annealing conditions of the reverse primer (RV) [19].

While the principal methods for obtaining ssDNA, including asymmetric PCR, magnetic separation with streptavidin-coated beads, lambda exonuclease digestion, and electrophoretic size separation, have been extensively reviewed [20], the authors were unable to identify a single method offering a distinct advantage. Experimental evaluation of magnetic separation with streptavidin-coated beads, enzyme digestion, and asymmetric PCR combined with enzyme digestion demonstrated advantages of the latter combination [21]. A subsequent comparative study revealed that electrophoretic separation after PCR with an extended reverse primer (RV) offered advantages over conventional magnetic separation using streptavidin-coated beads [22]. Given that these and other [6] results cast doubt on the effectiveness of magnetic separation with streptavidin-coated beads, we excluded this method from our study. We took into account recent developments for asymmetric PCR [17,23] and made a direct comparison of four most promising approaches for ssDNA generation in terms of efficiency, specificity, reproducibility, consume of time and reagents.

## 2. Experimental Design

### 2.1. Design of Study

**Figure 1:**
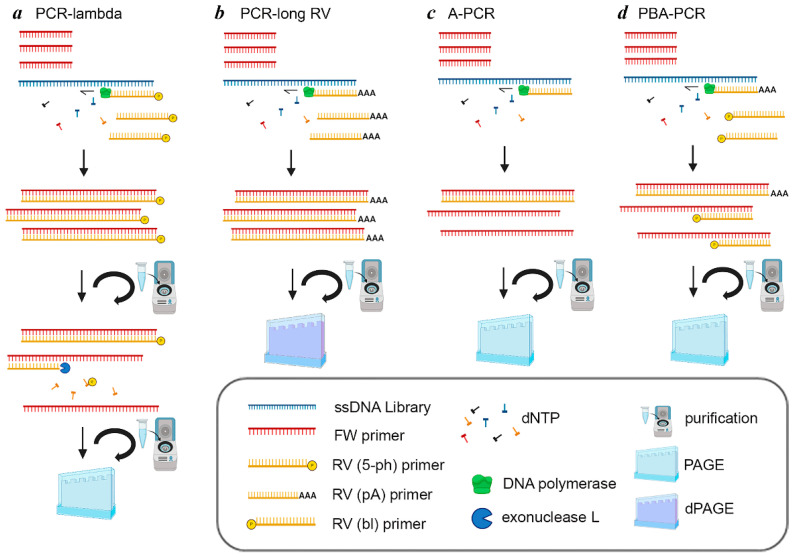
Scheme of ssDNA generation methods.

Four different approaches to generate ssDNA were optimized and performed in parallel, starting with the same amount of synthetic library: (1) symmetric PCR with a phosphorylated reverse primer (RV) followed by enzymatic cleavage of the reverse strand and ssDNA purification (hereafter referred to as PCR-lambda), (2) symmetric PCR with an extended reverse primer (RV) followed by denaturing separation of ssDNA (hereafter referred to as PCR-long RV), (3) asymmetric PCR (hereafter referred to as A-PCR), and (4) asymmetric PCR with a blocking and extended reverse primer (RV) (hereafter referred to as PBA-PCR). All methods are presented schematically in Figure 1.

### 2.2. Materials

- Chloroform (Lenreaktiv Ltd., Saint Petersburg, Russia, Cat.no.:210100);
- Phenol (Lenreaktiv Ltd., Saint Petersburg, Russia, Cat.no.:200121);
- Ammonium acetate (Lenreaktiv Ltd., Saint Petersburg, Russia, Cat.no.:010299);
- Isoamyl alcohol (Lenreaktiv Ltd., Saint Petersburg, Russia, Cat.no.: 010131);
- Ethylenediaminetetraacetic acid (EDTA); (Lenreaktiv Ltd., Saint Petersburg, Russia, Cat.no.: 600047);
- Urea (Lenreaktiv Ltd., Saint Petersburg, Russia, Cat.no.: 120427);
- Magnesium acetate tetrahydrate (Lenreaktiv Ltd., Saint Petersburg, Russia, Cat.no.: 120062);
- A 40% acrylamide/N,N’-methylenebisacrylamide 19:1 solution (Bio-Rad Laboratories, Hercules, CA, USA, Cat.no.: 1610145);
- Formamide (Khimmed Ltd., Saint Petersburg, Russia, Cat.no.: BP227-500);
- Sodium dodecyl sulfate and TBE ×10 buffer, pH 8.3 (Dia-M Ltd., Saint Petersburg, Russia, Cat.no.: LC-10112.1 and Cat.no.: 3774.0250);
- Ethidium bromide 10 mg/mL (Helicon Ltd., Saint Petersburg, Russia, Cat.no.: EtBr-10). Considering the high toxicity of ethidium bromide, alternative reagents (SYBR Green I/II, GelRed/GelGreen, Methylene Blue, Novel Juice) can be used instead;
- DNA marker Step50 plus and 4 × buffer for applying DNA to the gel “BiK” (both from Biolabmix Ltd., Novosibirsk, Russia, Cat.no.: S-8055), GeneRuler 50bp DNA Ladder Thermo Fisher Scientific, Waltham, MA, USA, Cat.no.: SM0372);
- Lambda phage exonuclease and 10× exonuclease reaction buffer (both from New England Biolabs, Frankfurt, Germany, Cat.no.: M0262S);
- Ethyl alcohol 96%;
- PCR-grade water (Biolabmix Ltd., Novosibirsk, Russia, Cat.no.: SP010-05),
- PCR master mix «BioMaster HS-Taq PCR x2» (Biolabmix Ltd., Novosibirsk, Russia, Cat.no.: MH010-200). Mix included 100 mM Tris-HCl, pH 8.5, 100 mM KCl, 0.4 mM of each dNTP, 4 mM MgCl_2_, 0.06 units of activity/μlTaq DNA polymerase and 0.2% Tween 20;
- Synthetic oligonucleotides (Syntol Ltd., Moscow, Russia). Sequences presented in Table 1. Phosphorylated (5′-end) reverse primers from various suppliers were used for comparative analysis.

### 2.3. Equipment

- DNA amplifier “BIS” M111-02-48 (Chaldin, Novosibirsk, Russia);
- MicroCL 17R Centrifuge (Thermo Fisher Scientific, Waltham, MA, USA);
- iBright™ FL1000 Imaging System (Thermo Fisher Scientific, Waltham, MA, USA);
- Vertical Electrophoresis Chamber VE-10-v2 (Helicon, Moscow, Russia);
- PowerPac^TM^ Basic Power Supply (Bio-Rad Laboratories, Hercules, NY, USA);
- Thermo shaker TS-100 (BioSan, Riga, Latvia);
- NanoPhotometer N50 spectrophotometer (Implen, Munchen, Germany).

## 3. Procedure

### 3.1. Polymerase Chain Reaction

Amplification was performed in 25 μL reactions containing 1 ng of ssDNA library in 1x BioMaster HS-Taq PCR buffer. Primer combinations and quantities varied depending on the PCR method (Table 2).

In the case of PBA-PCR, the amplification program included a preliminary denaturation step for 5 min followed by 25 cycles (denaturation for 10 s at 95 °C, annealing for 30 s at 60 °C and elongation for 20 s at 72 °C) and a final cycle of 72 °C for 30 s, while in other cases, the PCR conditions differed in shorter primer annealing (10 s at 60 °C) and elongation (10 s at 72 °C) times (Table 3).

### 3.2. dsDNA Extraction from the Reaction Mixtures

To purify and concentrate the PCR products, the reaction mixtures were pooled. An equal volume of phenol:chloroform:isoamyl alcohol mixture (25:24:1, *v*/*v*) was then added to the pooled PCR reactions, mixed vigorously, and centrifuged for 10 min in a refrigerated centrifuge at maximum speed (17,000× *g*, +4 °C). The aqueous fraction was collected, and 1/10 volume of 3 M ammonium acetate was added to it, followed by mixing. Next, 2.5 volumes of 96% ethyl alcohol were added to the mixture, and the mixture was then cooled at −80 °C for 1 h, and the precipitate was collected by centrifugation for 60 min (17,000× *g*, +4 °C). The supernatant was removed, and the sediment was washed twice with 70% ethyl alcohol and dried in air for 2 min. The precipitate was dissolved in PCR-grade water to a final volume of 40 μL and quantified using a NanoPhotometer N50 spectrophotometer (Implen GmbH, Munich, Germany) at a wavelength of 260 nm.

### 3.3. Exonuclease Digestion

Purified and concentrated dsDNA (1 µg) from PCR with a phosphorylated reverse primer (5′-ph) was incubated with 5 U of lambda exonuclease in 1x exonuclease reaction buffer in a volume of 50 µL for either 30 or 60 min at 37 °C. The reaction was stopped by enzyme inactivation at 75 °C for 10 min. The exonuclease reaction product was then purified and concentrated by phenol/chloroform extraction as described above.

### 3.4. Polyacrylamide Gel Electroforesis (PAGE)

PCR amplification products were analyzed by electrophoresis on a 6% polyacrylamide gel in 1× TBE buffer. Electrophoresis was performed at 120 V for 55 min. The gel was stained with 1× TBE containing 0.005% (*v*/*v*) ethidium bromide and then visualized using the iBright™ FL1000 gel imaging system. When necessary, bands containing the product were excised from the gel for further purification.

### 3.5. Denaturing PAGE (dPAGE)

Purified PCR amplification products were analyzed by denaturing polyacrylamide gel electrophoresis (dPAGE) containing 8 M urea (8% *w*/*v*) in 1× TBE buffer. Samples were pre-mixed with formamide in a 1:1 ratio, denatured at 95 °C for 5 min, and immediately cooled on ice before being dissolved in DNA loading buffer. Electrophoresis was conducted at 120 V for 70 min after pre-running the gel. The gel was stained with 1× TBE buffer containing 0.01% (*v*/*v*) ethidium bromide and then visualized using the iBright™ FL1000 gel imaging system. If necessary, bands containing the product were excised from the gel for further purification.

### 3.6. ssDNA Extraction from Polyacrylamide Gel

Amplification products were isolated from polyacrylamide gel using a modified Crush and Soak method [24]. Excised gel bands were crushed with a pipette tip in a test tube, and the crushed gel was then combined with 300 μL of elution buffer (10 mM magnesium acetate tetrahydrate, 0.5 M ammonium acetate, 1 mM EDTA, pH 8.0, and 0.1% (*w*/*v*) sodium dodecyl sulfate). The mixture was incubated at −80 °C for 1 h, followed by incubation in a thermo shaker TS-100 for 3 h at 55 °C with constant shaking. The supernatant was separated from the gel fragments by centrifugation (17,000× *g*) and transferred to a fresh tube. The ssDNA was purified by phenol/chloroform extraction as described above. The recovered precipitate was dissolved in 10 μL of PCR-grade water.

### 3.7. Statistical Analysis

The specificity of symmetric PCR was assessed by measuring the intensity of the FAM-labelled dsDNA amplicon band on PAGE using densitometric analysis with ImageJ (Version 1.54). Similarly, the efficiency of asymmetric PCR was evaluated by measuring the intensity of the FAM-labelled ssDNA band, which migrates faster than dsDNA. For each sample, the total pixel value of each line was normalized to 100%, allowing us to determine the percentage pixel value representing the specific product. Results obtained after 10, 15, 20, and 25 cycles were averaged to facilitate a general comparison of the four methods.

The efficiency of each method was estimated by quantifying the purified ssDNA yielded from ten reactions performed in 25 μL, using 1 ng of the ssDNA library. All experiments were conducted in triplicate, and the results were expressed as the mean. The reproducibility of the methods was assessed by calculating the root mean square deviation (RMSD) of the ssDNA yields:

$$\left(RMSD=\sqrt{\frac{\sum_{i=1}^{n}{\left(xi-\bar{x}\right)}^{2}\;}{n-1}},\;where\;is\;\bar{x\;}-the\;average\;value\right)$$

Reproducibility of each method was evaluated via the Variation Coefficient (V):

$$V=\frac{RMSD}{\bar{x}}$$

## 4. Protocol Setup

### 4.1. Specific Aspects of PCR-Lambda

The success of the PCR-lambda method (symmetric PCR with a phosphorylated reverse primer, RV(5′-ph), followed by enzymatic cleavage of the reverse strand, as illustrated in Figure 1a was largely dependent on the efficacy of exonuclease digestion. Optimization of the reaction conditions (dsDNA amount, incubation time, and enzyme concentration) is critical, as described previously [10].

**Figure 2:**
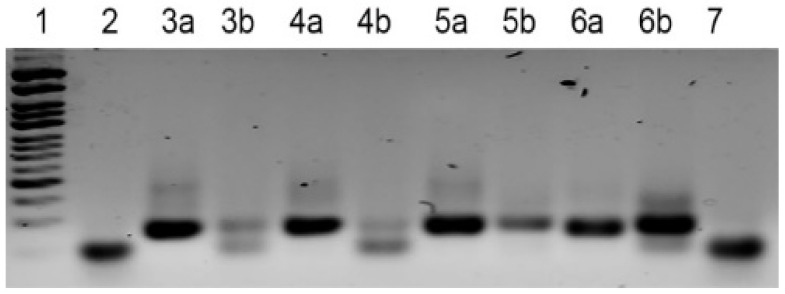
The impact of RV(5′-ph) primer quality on the efficiency of exonuclease digestion in the PCR-lambda method was investigated. Four parallel PCR reactions were performed, differing only in the source of the RV(5′-ph) primer. The amplification products were purified, and 1 µg of dsDNA from each reaction was digested by lambda exonuclease for 30 min. The products were then separated on a 3% agarose gel Line 1—Step50 plus DNA marker and for lines 2 and 7, ssDNA library used for PCR with a size equal to the expected ssDNA product of exonuclease reaction. The lines 3, 4, 5 and 6 are the results of experiments carried out with different RV(5′-ph). In each pair of lines: a—dsDNA PCR product (≈20 ng); b—product of exonuclease reaction (≈60 ng) made from a mixture of dsDNA and ssDNA.

We designed an experiment to evaluate the influence of phosphorylated primer quality on the performance of exonuclease digestion. Four parallel PCR amplifications were performed, each followed by exonuclease digestion. The RV(5′-ph) primers used in these reactions were sourced from four distinct suppliers. As shown in Figure 2, the quality of the RV(5′-ph) primer significantly affected the digestion process. Consequently, the primer exhibiting the highest digestion efficiency (Figure 2, lane 4b) was chosen for all subsequent experiments.

### 4.2. Specific Aspects of PCR-Long RV

A distinctive feature of the PCR method employing an extended reverse primer, followed by ssDNA separation using denaturing polyacrylamide gel electrophoresis (dPAGE) (PCR-long RV, Figure 1b), is the structure of the extended 5′ end of the reverse primer RV(A). In this study, the primer sequence was supplemented with a C18 spacer (hexaethylene glycol chain, 18 atoms long) and a non-replicating poly(dA)_20_ tail (Table 1). However, other structures can be effective as well. The PCR conditions were the same as those for using an unmodified reverse primer. Then, the unequal chains of the obtained dsDNA were separated in denaturing PAGE and short chains were excised and purified.

### 4.3. Specific Aspects of A-PCR and PBA-PCR

The most critical factor in establishing asymmetric PCR (A-PCR, Figure 1c) is the primer concentration ratio (FW:RV). We tested different ratios, and found that 20:1 was the best ratio. However, considering the possible impact of other factors, it was recommended to test this parameter experimentally.

**Figure 3:**
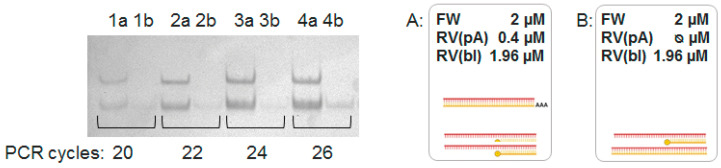
Electrophoresis in 6% PAGE of PBA-PCR amplification products. Lanes 1a, 2a, 3a and 4a correspond to 20, 22, 24 and 26 cycles of PCR with all primers: FW, RV(pA) and RV(bl) as shown in scheme (**A**). Lanes 1b, 2b, 3b and 4b correspond to 20, 22, 24 and 26 cycles of PCR with only FW and RV(bl) primers as shown in scheme (**B**).

A modified version of asymmetric PCR (PBA-PCR, Figure 1d) employed equal amounts of forward (FW) and reverse primers, where the reverse primers comprised a mixture of blocking, RV(bl), and extended, RV(pA), primers. The RV(pA) primer initiated synthesis of a limited amount of the heavy antisense strand, which served as the initial template for single-stranded DNA synthesis. The presence of the poly(dA)_20_ tail facilitated separation of the PBA-PCR product mixture on polyacrylamide gel electrophoresis (PAGE). The 3′-phosphorylated RV(bl) primer annealed to the primer landing site on the sense strand and blocked synthesis of a complementary, unwanted strand. In order to check if RV(bl) indeed prevents formation of undesired antisense strands, we performed PBA-PCR without an RV(pA) primer. Figure 3 shows the products of both control (A) and experimental (B) reactions after 20, 22, 24 and 26 cycles of PCR. Our interpretation is that the 5′-phosphorylated reverse primer RV(bl), while intended as a complete block to extension, exhibits a low level of incomplete termination. The occasional extension from this non-phosphorylated RV would then create a binding site for the forward primer (FW), enabling subsequent amplification, albeit at a reduced rate. Given that this effect is only apparent after 26 cycles, it likely plays a negligible role in the overall PCR process.

The optimal primer ratio was experimentally determined to be 50:1:49 (FW:RV(pA):RV(bl)). Purification of ssDNA from double-stranded amplicons was achieved, as in classical asymmetric PCR, by PAGE followed by extraction of the ssDNA from the gel.

## 5. Results and Discussion

### 5.1. Evalution of PCR Specificity

**Figure 4:**
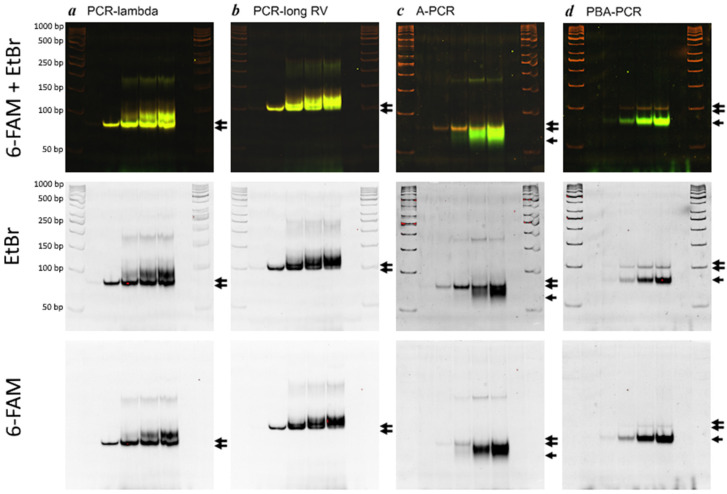
Electrophoresis in 6% PAGE of amplification products obtained at consequent cycles by the four methods tested. (**a**) PCR-lambda; (**b**) PCR-long RV; (**c**) A-PCR; (**d**) PBA-PCR. Lines 1 and 8: DNA Ladder (GeneRuler 50 bp). Gels were imaged on an iBright FL1000 imaging system in fluorescent blot imaging mode using the FITC and Qdots 605 channels. Lines 2, 3, 4, 5, and 6 correspond to the PCR cycle numbers 5, 10, 15, 20 and 25 at which the amplification was completed. Line 7: template-free control. Double arrows indicate dsDNA products; single arrows indicate ssDNA products.

During the SELEX procedure, preparative PCR is typically performed until a critical amount of byproduct appears. Therefore, we sought to determine the optimal number of PCR cycles that would provide the best ratio between the correct amplicon and nonspecific byproducts. PCR reactions were performed under the conditions described previously using a forward primer FW(FAM) labeled 6-carbofluorescein. The reactions were terminated, and the tubes removed, after 5, 10, 15, 20, and 25 cycles. The amplification products were then separated by electrophoresis on a 6% PAGE gel. Figure 4 illustrates the dynamic changes in the composition of amplification products, which varied across the four methods.

**Figure 5:**
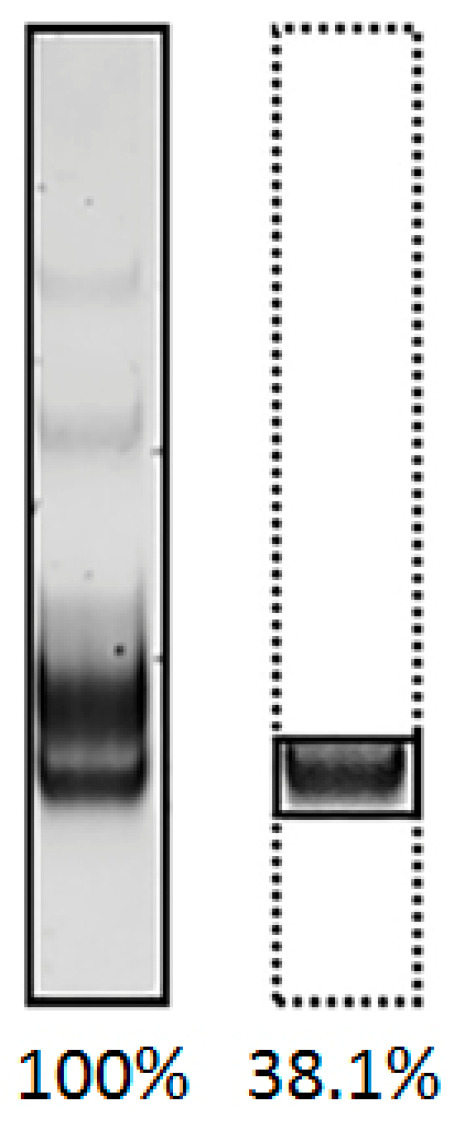
Principle of assessment of PCR specificity with ImageJ software. The total pixel value of the line was assumed to be 100%, and the area containing a specific product was estimated as a percentage.

This experiment revealed relatively high efficacy but low specificity for standard (symmetric) PCR (Figure 4: A and B vs. C and D). The high efficacy can be attributed to the exponential mode of amplification. The increase in byproducts may be due to primer annealing within the random sequence region and the gradual accumulation of extended amplicons. Furthermore, both the PCR-lambda and PCR-long RV methods produced two additional bands of distinct sizes: 200 bp and 250 bp for PCR-lambda(6-FAM); 250 bp and 400 bp for PCR-long RV(6-FAM). These may represent complexes of two or three amplicons. In contrast to symmetric PCR, both approaches to asymmetric PCR (A-PCR, PBA-PCR) demonstrated increased specificity. To compare the specificity of each method, the relative amounts of the desired PCR products were quantified using ImageJ software. An example of this approach is shown in Figure 5. The presence of a fluorescent label on all amplification products in each method enabled us to compare the relative amounts of dsDNA and ssDNA while minimizing the impact of their differing affinities for ethidium bromide.

Table 4 presents the percentages of the specific product obtained by each method after 10, 15, 20 and 25 PCR cycles. Despite the fact that the PCR-lambda and PCR-long RV methods produced dsDNA, while the A-PCR and PBA-PCR methods produced ssDNA, the relative amount of the specific product reflected the specificity of each approach. The highest specificity was observed with PCR-lambda and PCR-long RV after 10 PCR cycles and with A-PCR and PBA-PCR after 25 PCR cycles. The average values (last row of Table 4) were calculated to facilitate a general comparison of the four methods’ specificity.

### 5.2. Evaluation of Efficacy of ssDNA Generation

Based on the dynamic ratio of product to byproduct, the optimal number of cycles for preparative PCR was selected for each method: 10 cycles for the PCR-lambda and PCR-long RV methods, and 25 cycles for the A-PCR and PBA-PCR methods (Figure 2 and Table 4).

**Figure 6:**
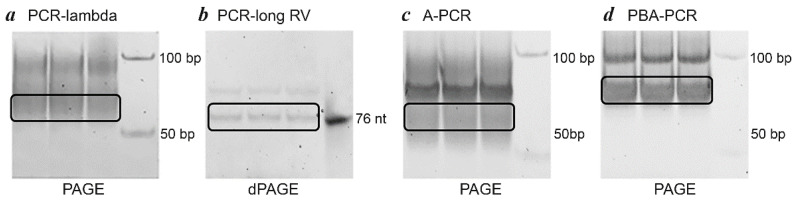
Generation and separation of ssDNA by electrophoresis in 6% PAGE. The images show products generated by different methods from the same amount of ssDNA. In all cases, lines 1–3 contain the PCR product combined from 10 reactions, line 4 includes either Step50 plus DNA marker ((**a**,**c**,**d**) in PAGE) or ssDNA library ((**b**) in dPAGE). Bands containing ssDNA, indicated by frames, were excised for further purification.

After selecting the optimal number of PCR cycles, preparative PCR was performed using each of the four methods. The reaction mixtures (25 µL × 10) were combined to yield a total volume of 250 µL. The PCR products were purified by phenol/chloroform extraction and dissolved in 10 µL of water. For the PCR-lambda method (Figure 1a), the dsDNA PCR product was subsequently treated with lambda exonuclease, followed by additional purification. Purified DNA was loaded into gel (3.3 µL per line) and separated by electrophoresis. The products of the PCR-lambda, A-PCR, and PBA-PCR methods were separated by PAGE (Figure 6a,c,d), while the product of the PCR-long RV method (Figure 6b) was separated by denaturing PAGE (dPAGE).

Next, the fragments of the gel containing ssDNA were excised as shown in Figure 6. The amounts of isolated ssDNA, reflecting the overall efficiency of each method, were quantified by spectrophotometry at a wavelength of 260 nm. Each of the four experiments were performed in triplicate and the results of three independent assays were averaged. All results are summarized in Table 5. The PBA-PCR method demonstrated the highest yield (553.6 ± 93.7 ng), while the A-PCR method was less effective (191.4 ± 16.8 ng) and a relatively low amount of ssDNA was generated by PCR-lambda (110.5 ± 33.6 ng) and PCR-long RV (88.2 ± 30.6 ng).

**Figure 7:**
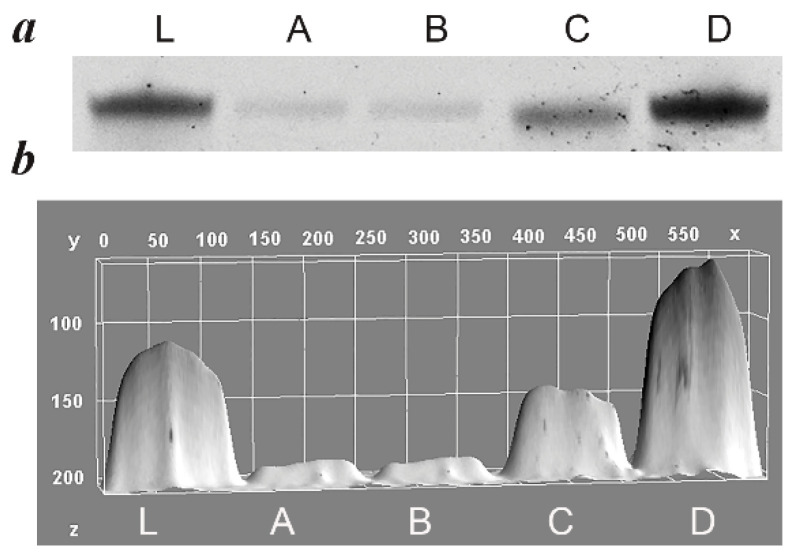
Quantity ssDNA generated by four different methods from the same amount of ssDNA (3 × 10 PCR reactions). Products of PCR were pooled, ssDNA was purified by corresponding approaches and dissolved in 10 μL of water and 3 μL of ssDNA solution was loaded into denaturating gel for electrophoresis. (**a**) Fragment of denaturing 6% PAGE; (**b**) pixel density of bands quantified by ImageJ software. In both images, lines correspond to 100 ng library DNA (L), ssDNA generated by PCR-lambda (A), PCR-long RV (B), A-PCR (C) and PBA-PCR method (D).

In the case of PBA-PCR, the amplicons were annealed with the blocking primers RV(bl); therefore, the estimated concentration reflected the combined amount of ssDNA and blocker. To confirm differences in ssDNA abundance, the products were further separated by denaturing PAGE (dPAGE), where the visualized bands reflected the amount of pure ssDNA product (Figure 7).

As shown in Figure 7a, all products had the expected size, corresponding to the size of the ssDNA library (lane L). The dPAGE results revealed considerable differences in the efficacy of the four methods tested, confirming the results of spectrophotometry.

### 5.3. Reproducibility and Feasibility of Methods

In addition, we assessed the convenience of each method based on three criteria: reproducibility, time consumption, and specific reagent requirements (Table 5). Reproducibility and time consumption were estimated from three independent experiments for each of the four methods. Thus, A-PCR could be performed within 8–9 h; the PBA-PCR and PCR-long RV methods required more time, whereas PCR-lambda, including an additional enzymatic reaction and purification steps, required almost 12 h to complete. The reproducibility of the methods was determined by calculating the root mean square deviation (RMSD) of the ssDNA yields and variation coefficient (V). A higher V value indicates lower reproducibility regardless of the magnitude of the mean value. Considering this parameter, the reproducibility of the methods increases in the following order: PCR-long RV, PCR-lambda, PBA-PCR and A-PCR.

Table 5 summarizes the characteristics of the four methods tested, including the specific reagents required.

## 6. Conclusions

Herein, we compared four methods of ssDNA generation used in the context of SELEX technology. The PBA-PCR yielded the largest amount of ssDNA compared to the other methods tested and also exhibited the highest percentage of specific PCR products. However, the reproducibility of the ssDNA yield varied across experiments. The A-PCR method produced a lower ssDNA yield than PBA-PCR and had the lowest percentage of specific PCR products, but its excellent reproducibility, relatively short turnaround time, and low cost (no specific reagents) were advantages of this approach. The PCR-long RV method showed the lowest ssDNA yield, and its performance in terms of other parameters was also unremarkable. The PCR-lambda method demonstrated a relatively low yield of ssDNA, moderate specificity and reproducibility, and it suffered from the greatest labor intensity and the need for specific reagents. The obtained results revealed the best characteristics of PBA-PCR methods that can be effectively implemented in the SELEX procedure. The detailed protocol of PBA-PCR in numbered bulleted format can be obtained from Supplementary File S1.

The primary value of this work lies in the direct comparison of four different methods and the experimental confirmation of the advantages of PBA-PCR. We performed a comparative analysis based on several important criteria, demonstrating that PBA-PCR is the superior approach when considering a comprehensive analysis. In addition, we have thoroughly documented the limitations, or “pitfalls,” of the other methods (PCR-lambda, PCR-long RV, A-PCR). This detailed information may be useful for researchers seeking to further optimize any of the methods that yielded suboptimal results in our experiment.

## Figures and Tables

**Table 1:** Primers and oligonucleotides used in PCR.

Name	Structure 5′→3′	Length, nts
ssDNA library *	TAGGGAAGAGAAGGACATATGAT-(N)30-TTGACTAGTACATGACCACTTGA	76
FW	TAGGGAAGAGAAGGACATATGAT	23
FW(FAM)	[6-FAM]-TAGGGAAGAGAAGGACATATGAT	23
RV	TCAAGTGGTCATGTACTAGTCAA	23
RV(5′-ph)	phosphate-TCAAGTGGTCATGTACTAGTCAA	23
RV-poly(dA)	AAAAAAAAAAAAAAAAAAAA(spacer18)TCAAGTGGTCATGTACTAGTCAA	43
RV(bl)	TCAAGTGGTCATGTACTAGTCAA-phosphate	23

* A 76-nucleotide-long ssDNA library was used as a template for PCR, in which the central region of 30 nucleotides was randomized and flanked on both sides by landing sites for 23-nucleotide-long primers.

**Table 2:** Concentration of primers in reaction mixture used at different PCR methods.

Primers	PCR-lambda	PCR-long RV	A-PCR	PBA-PCR
FW/FW(FAM)	2 µM	2 µM	1.6 µM	2 µM
RV(5′-ph)	2 µM	-	-	-
RV(pA)	-	2 µM	-	0.04 µM
RV	-	-	0.08 µM	-
RV(bl)	-	-	-	1.96 µM

**Table 3:** Conditions of different PCR methods.

Stage		PCR-Lambda PCR-Long RV A-PCR	PBA-PCR
First denaturation		95°C—5′	95°C—5′
Denaturation	PCR	95°C—10″	95°C—10″
Annealing		60°C—10″	60°C—30″
Elongation		72°C—10″	72°C—20″
Final elongation		72°C—30″	72°C—30″

**Table 4:** Relative quantity of specific product yielded by different methods, %.

Cycles	PCR-lambda	PCR-long RV	A-PCR	PBA-PCR
10	100	100	48.6	100
15	68.7	40.1	53.0	89.7
20	49.8	22.0	69.3	90.5
25	38.1	13.4	71.7	90.5
Average	51.3	35.1	48.5	92.7

**Table 5:** Comparison of parameters of different ssDNA generation methods.

	PCR-lambda	PCR-long RV	A-PCR	PBA-PCR
Specificity (relative amount of specific product, %)	51.3	35.1	48.5	92.7
Efficacy (yield of purified ssDNA ± RMSD, ng)	110.5 ± 33.6	88.2 ± 30.6	191.4 ± 16.8	553.6 ± 93.7
Variation Coefficient, V	0.31	0.35	0.09	0.17
Time consumed (protocol duration, hours)	11.5	9.5	8.5	9.0
Specific reagents	Lambda Exonuclease; RV(5′-ph)	RV(dA); dPAGE	-	RV(dA); RV(bl)

## Data Availability

No new data were created within the study.

## Supplementary Materials

The following supporting information can be downloaded at: www.mdpi.com/article/10.3390/mps8020036/s1, File S1: Protocol generation of ssdna using PBA-PCR method.

## Abbreviations

The following abbreviations are used in this manuscript:

SELEX	Systematic evolution of ligands by exponential enrichment
ssDNA	Single-stranded deoxyribonucleic acid
dsDNA	Double-stranded deoxyribonucleic acid
PCR	Polymerase chain reaction
PAGE	Polyacrylamide gel electrophoresis
dPAGE	Denaturing urea polyacrylamide gel electrophoresis
TBE buffer	Tris-borate-EDTA buffer
A-PCR	Asymmetric polymerase chain reaction
PBA-PCR	Primer-blocked asymmetric polymerase chain reaction
