# Supplementary material for: Specific Aspects of SELEX Protocol: Different Approaches for ssDNA Generation
Source: Methods Protoc. 2025 Apr 6;8(2):36. doi: 10.3390/mps8020036 (PMC12029403; doi:10.3390/mps8020036)
Supplement: Supplementary file 1 [file mps-08-00036-s001.zip › mps-3504762-supplementary.pdf]

Specific aspects of SELEX protocol: different approaches for ssDNA generation

Alexandr Garanin, Andrey Shalaev, Lidia Zabegina, Ekaterina Kadantseva, Tatiana Sharonova, Anastasia Malek*

PROTOCOL GENERATION OF SSDNA USING PBA-PCR METHOD

1. Amplification of ssDNA libraries

Reagents and Materials

- 10 x PCR buffer and Thermostable Hot Start Taq DNA polymerase;
- 25 mM dNTP mix;
- Deionized or Milli-Q water (18.2 MΩ·cm);
- Pipette tips with filters;
- 1.5-ml DNA LoBind tubes (Eppendorf, cat. no. 022431021);
- 0.2-ml PCR tubes (or 96-well PCR plate);
- Primers and oligonucleotides(Table S1).

Table S1. Primers and oligonucleotides used in PBA-PCR

Name	Structure 5′ → 3′	Length, nts
DNA library	TAGGGAAGAGAAGGACATATGAT-(N)30-TTGACTAGTACATGACCACTTGA	76
FW	TAGGGAAGAGAAGGACATATGAT	23
RV(pA)	AAAAAAAAAAAAAAAAAAAA(spacer18)TCAAGTGGTCATGTACTAGTCAA	43
RV(bl)	TCAAGTGGTCATGTACTAGTCAA-phosphate	23

Equipment

- Thermocycler for PCR.

Protocol

1. Defrost thermostable DNA polymerase and buffer, dNTPs, primers, template DNA library, and deionized water. Keep all reagents and samples at 4°C on ice.
2. Prepare the following dilutions of primers using deionized water and DNA LoBind tubes:
    - 10 μM FW primer;
    - 1 μM RV(pA) primer;
    - 10 μM RV(bl) primer.
3. Mix the following components in a clean PCR tube for each 25-μl PCR mixture:

- 2.5 µl 10× PCR buffer (final 1×);
- 5 µl 10 µM FW primer (final 2 µM);
- 1 µl 1 µM RV(pA) primer (final 0.04 µM);
- 4.9 µl 10 µM RV(bl) primer (final 1.96 µM);
- 0.2 µl 25 mM dNTP mix (final 0.2 mM each);
- 0.2 µl 5 U/µl Thermostable Hot Start Taq DNA polymerase (final 0.04 U/µl);
- 1 µl 10 nM DNA library (final 0.8 µM) or deionized water in case no-template control(NTC);
- 10.2 µl deionized water.

4. Place the tube(s) in the thermal cycler and run the program listed in Table S2.

Table S2. Conditions of PBA-PCR

Stage		PBA-PCR	Number of PCR cycles
First denaturation		95°C – 5′	1
denaturation	PCR	95°C – 10″	25
annealing		60°C – 30″	
elongation		72°C – 20″	
Final elongation		72°C – 30″	1

2. Phenol-chloroform extraction

Reagents and Materials

- Chloroform;
- Fenol;
- Ammonium acetate;
- Isoamyl alcohol;
- Ethyl alcohol 96%;
- Deionized or Milli-Q water (18.2 MΩ·cm);
- Pipette tips with filters;
- 1.5-ml DNA LoBind tubes (Eppendorf, cat. no. 022431021).

Equipment

- MicroCL 17R Centrifuge, Refrigerated (Thermo Fisher Scientific, Waltham, USA).

Protocol

1. Combine the contents of at least 10 PCR tubes(except NTC tube) to obtain a sample of at least 250 μl;
2. Add one volume of phenol:chloroform:isoamyl alcohol (25:24:1, 125μl:120μl:5μl) to your sample, and shakeby hand thoroughly for approximately 30 seconds;
3. Centrifuge for 10 min in a refrigerated centrifuge at maximum speed (17000g and +4°C).Carefully remove the upper aqueous phase, and transfer the layer to a fresh tube;
4. Add to the aqueous fraction 1/10 of the volume of 3 M ammonium acetate, mixed. Then 2.5 parts of 96% ethyl alcohol were added to the mixture, place the tube in –80°C for at least 1 hour;
5. Centrifuge the sample at 4°C for 60 minutes at maximum speed to pellet the dsDNA/ssDNA;
6. Carefully remove the supernatant without disturbing the cDNA pellet;
7. Add 150 μL of 70% ethanol. Centrifuge the sample at 4°C for 2 minutes at maximum speed. Carefully remove the supernatant;
8. Repeat Step 7 once. Remove as much of the remaining ethanol as possible;
9. Dry the dsDNA/ssDNA pellet at room temperature for 5–10 minutes;
10. Resuspend the dsDNA/ssDNA pellet in 10 μL of deionized water by pipetting up and down;
11. Centrifuge briefly to collect the sample, and place the tube on +4C.

3. Purification of ssDNA

Reagents and Materials

- TBE x10 buffer, pH 8.3;
- 40% acrylamide/N,N′-methylenebisacrylamide 19:1 solution;
- Elution buffer;
- Ammonium persulfate (APS), 30% (w/v);
- TEMED (CAS 110-18-9);
- Ethidium bromide or GelRed nucleic acid stain (Biotium, cat. no. 41003, USA);
- DNA Gel Loading Dye (6X)(Thermo Fischer, cat. no.R0611, USA)
- GeneRuler Low Range DNA Ladder, ready-to-use (Thermo Fischer, cat. no.SM1193, USA);
- Pipette tips with filters;
- Nunc 15 ml Conical Sterile Polypropylene Centrifuge Tubes (Thermo Fischer, cat. no.339651, USA).

Equipment

- MicroCL 17R Centrifuge, Refrigerated (Thermo Fisher Scientific, Waltham, USA);
- iBright™ FL1000 Imaging System (Thermo Fisher Scientific, Waltham, USA);
- Vertical Electrophoresis Chamber;
- PowerPacTM Basic Power Supply (Bio-Rad Laboratories, Hercules, USA);
- Thermo shaker TS-100 (BioSan, Riga, Latvia);
- Spectrophotometr Nano Drop 2000 (Thermo Fisher Scientific, Waltham, USA);

Solutions to be prepared

- Ammonium persulfate (APS), 30% (w/v)

  0.3 g ammonium persulfate (CAS 7727-54-0)

  0.7 ml deionized water

  Store up to 6 months at −20°C

- Tris-borate-EDTA (TBE) buffer, 5×

  60.55 g Tris base (CAS 77-86-1)

  30.9 g boric acid (CAS 10043-35-3)

  3.7 g Ethylenediaminetetraacetic acid disodium salt dihydrate (EDTA) (CAS 6381-92-6)

  Adjust volume to 1000 ml with deionized water

  Store up to 2 months at room temperature

- GelRed staining solution, 1×

  5 µl 10,000× GelRed dye (Biotium, cat. no. 41003)

  50 ml deionized water or 1×TBE

  Store in the dark for up to 1 week at 4°C

- Elution buffer

  0.2g SDS (CAS 151-21-3)

  0.2 ml 0,5M EDTA, pH 8,0

3.9g Ammonium acetate (CAS 631-61-8)

0.15g Magneum acetate (CAS 16674-78-5)

Store up to 3 months at room temperature

Protocol

1. Prepare the electrophoresis apparatus. Assemble cleaned and degreased glass plates with 1.0-mm spacers and fix them in the holder.
2. Thaw frozen 30% (w/v) APS at room temperature;
3. Mix the first three components of a 6% gel mixture in a clean conical tube:

- 6.5 ml deionized water;
- 1.5 ml 40% acrylamide/bis-acrylamide solution (19:1);
- 2 ml 5× TBE buffer.

4. Add 33 μl APS and 4 μl TEMED to the gel solution.Mix immediately and thoroughly by pipetting and pour between the two plates;
5. Insert the comb to create the holes. Allow the gel to cure for 0.5 hour;
6. Mix samples with 6× DNA Gel Loading Dye in microcentrifuge tubes;
7. Disassemble the gel holder and carefully remove the comb from the gel. Fix the polymerized gel in the electrophoresis block. If you have only one gel, insulate the electrodes with a blank plate;
8. Fill the chambers with 1× TBE buffer, making sure it completely covers the wells and contacts both electrodes;
9. Load the samples(including NTC) and GeneRuler Low Range DNA Ladder into the gel wells. Start electrophoresis at 10-15 V/cm of gel length. Voltage to be applied (V) = gel length (cm) × recommended V/cm. For a 10 × 10 cm gel with a 1 mm pad, use a constant voltage of 120 V for 60 min;
10. Remove gel from the plates and place in GelRed staining solution;
11. Shake gently for 10-15 min;
12. Check NTC for absence of DNA bands, detect ssDNA product at 75 bp using DNA ladder, cut out band using blade, transfer to sterile tube;
13. Using a sterile tip, crush the gel, add 300 μl of elution buffer, place the tube at -80 C for 1 hour;
14. Incubate in a TS-100 Thermo-Shaker for 3 hours at 55 °C with constant shaking (800 rpm). Separate the supernatant from the gel fragments by centrifugation (17,000 g) and transfer to a sterile tube. Purify ssDNA by chloroform/phenol extraction as described above;
15. Measure the concentration of ssDNA using spectrophotometr.
